# Improving Antimicrobial Stewardship Program Using the Lean Six Sigma Methodology: A Descriptive Study from Mediclinic Welcare Hospital in Dubai, the UAE

**DOI:** 10.3390/healthcare11233048

**Published:** 2023-11-27

**Authors:** Mohammed Sallam, Johan Snygg

**Affiliations:** 1 Department of Pharmacy, Mediclinic Welcare Hospital, Mediclinic Middle East, Dubai P.O. Box 31500, United Arab Emirates; 2 Department of Pharmacy, Mediclinic Parkview Hospital, Mediclinic Middle East, Dubai P.O. Box 51122, United Arab Emirates; 3 Mediclinic Welcare Hospital, Mediclinic Middle East, Dubai P.O. Box 31500, United Arab Emirates; johan.snygg@mediclinic.ae; 4 Sahlgrenska Academy, The University of Gothenburg and Sahlgrenska University Hospital, 41345 Gothenburg, Sweden

**Keywords:** antimicrobial stewardship, quality improvement, compliance, quality of care, healthcare management, health policy

## Abstract

Background: Antimicrobial resistance (AMR) is one of the key challenges in healthcare, and effective antimicrobial stewardship programs (ASPs) can play a vital role to control it. The aim of the current study was to assess the impact of the Lean Six Sigma (LSS) methodology on a hospital-wide ASP. Methods: This retrospective descriptive study was conducted at Mediclinic Welcare Hospital (MWEL), Dubai, the United Arab Emirates (UAE). The critical-to-quality (CTQs) data were collected pre/post interventions, including leadership support, guideline implementation, and audits. The study focused on inpatients who received antimicrobials from January 2021 to July 2022, analyzing ASP metrics (utilization, processes, and outcome measures). Results: The ASP improvements led to an 81.7% decrease in hospital’s parenteral antimicrobial expenses from January 2021 to July 2022, and a 54.2% reduction in antimicrobial usage while maintaining clinical outcomes. The average defined daily dose per 100 bed-days drop of 12.5% further demonstrated this positive trend. The intervention was not accompanied by higher nosocomial infection rates, longer stays, or mortality. Additionally, intervention led to better compliance with surgical prophylaxis bundles, antimicrobial protocols, hand hygiene and other ASP CTQ metrics. Conclusions and recommendations: This study emphasized the significance of implementing the LSS methodology in addressing process variations, enhancing ASP outcomes, and reducing antimicrobial use. These findings can inform health policymakers to improve future ASP outcomes. Additionally, sustainability through continuous monitoring and ongoing education initiatives should be considered to ensure the long-term success of these improvements.

## 1. Introduction

The United Arab Emirates (UAE) healthcare structure, aligned with the country’s Vision 2021 National Agenda, seeks to achieve world-class standards by enhancing patient care quality [1]. Enhancing quality in the healthcare sector entails the adoption of high-reliability quality principles to transform process involved in healthcare [2].

Lean Six Sigma (LSS), a combined methodology, targets efficiency improvement by eradicating waste (Lean) and minimizing variation through data-driven defect reduction (Six Sigma). The LSS approach gained prominence in healthcare over the past two decades to help in optimizing operations and improving patient outcomes [3,4,5]. The Define, Measure, Analyze, Improve, Control (DMAIC) approach, integral to LSS, offers a structured problem-solving and enhancement framework [6].

A major challenge to healthcare systems causing global health concerns is the issue of antimicrobial resistance (AMR) [7]. Effective measures to properly address the rising AMR involve robust antimicrobial stewardship programs (ASPs), as recommended by the Centers for Disease Control and Prevention (CDC) and the Joint Commission International (JCI) [8,9,10]. The ASPs include interventions to optimize the use of antimicrobials, promote proper prescribing practices, and curb the emergence and spread of AMR; however, these interventions vary based on the organization size, resources, and extent of AMR as a healthcare challenge [11].

To ensure safer healthcare practices and better patient outcomes, the implementation of ASPs appears as a key measure [12,13]. The ASPs can guide the judicious use of antimicrobials; thus, preserving the efficacy of these treatments in the future [14]. Additionally, ASPs can contribute to patient safety by minimizing the risk of adverse effects associated with antimicrobial therapy and reducing the risk of emergence of multidrug resistant (MDR) microorganisms [15]. Moreover, ASPs can play an important role in identifying and preventing harmful drug interactions, especially in patients receiving multiple medications [16,17]. Furthermore, the implementation of ASPs can help to prevent unnecessary antimicrobial use with the subsequent optimization of resource allocation in healthcare [18].

Mediclinic Welcare Hospital (MWEL)—part of Mediclinic Middle East—is a well-established JCI-accredited healthcare institution in Dubai, the UAE [19]. Central values for MWEL encompass a patient-centric approach, emphasizing health and safety while adhering to elevated standards of service through ethical principles [20].

In 2021, an MWEL ASP intervention measure based on the DMAIC approach was implemented for antimicrobial stewardship. The rationale for employing the LSS methodology was related to several key considerations as follows: First, the efficiency of LSS can help establish a more efficient and streamlined management of antimicrobials [21]. Second, the LSS methodology can help identify gaps where the quality of an ASP can be enhanced [22]. Third, based on the dynamic nature of ASPs and the evolving nature of AMR, the LSS provides a dynamic framework for continuous improvement and enhances the ability to address the continuous challenge of AMR [7]. Finally, the LSS approach provides an optimal opportunity for optimizing the allocation of resources within ASPs, ensuring the effective utilization of these resources [23].

The primary objective of this quality improvement initiative was to reduce annual antimicrobial usage and expenditure on antimicrobial agents by at least 20%. The decision to conduct this project was based on the MWEL’s ongoing commitment to optimize antimicrobial use, based on the continuous quality improvement efforts to streamline antimicrobial prescribing, reduce unnecessary usage, and enhance patient outcomes. Additionally, MWEL aimed to investigate the impact of this intervention on ten parameters as follows: (1) parenteral antimicrobial utilization [24]; (2) utilization of high-risk agents like injectable fluoroquinolones (ciprofloxacin, levofloxacin, and moxifloxacin) and third-generation cephalosporins (ceftriaxone, cefotaxime, and ceftazidime) [25,26]; (3) restricted antimicrobial agent utilization [27,28]; (4) best surgical prophylaxis practice [29]; (5) conversion of intravenous (IV) to oral (PO) antimicrobials [30]; (6) compliance with infection control practices (hand hygiene, environmental cleaning, and patient cleanliness) [31,32,33]; (7) infectious disease (ID) physicians’ role [34]; (8) rate of infections caused by MDR microorganisms [35]; (9) average length of hospital stay [36]; (10) clinical pharmacist ASP intervention and reduction in adverse drug events [37].

Thus, this retrospective, observational descriptive study aimed to evaluate the impact of LSS methodology, specifically the DMAIC approach, on the effectiveness of the hospital-wide ASP at MWEL in Dubai, the UAE. This objective was pursued through the collection of critical-to-quality (CTQs) data on antimicrobial use practices before and after implementing nine ASP interventions. These interventions included: (1) leadership support; (2) guidelines for antimicrobial use; (3) formulary restriction with prior authorization; (4) utilization audits; (5) use of local antibiogram and compliance with culture and susceptibility results; (6) conversion of IV to PO antimicrobials; (7) assessment of antimicrobial use: appropriate selection, dosing, route, and duration; (8) feedback/education; and (9) proper cleaning and hand hygiene [38,39].

By employing the DMAIC approach, this study aimed to identify areas for improvement, implement targeted interventions, and assess the resulting changes in ASP metrics [40].

## 2. Materials and Methods

### 2.1. Study Setting and Design

A retrospective, observational descriptive study design was employed to assess the quality improvement initiative at MWEL, including various departments and services such as medicine, surgery, intensive care unit (ICU), neonatal, pediatrics, laboratory, radiology and pharmacy departments.

The study examined the antimicrobial utilization at two time points as follows: (1) the baseline period (before April 2021); (2) the intervention period (April 2021 to July 2022), marked by the implementation of an ASP initiative led by a multidisciplinary team.

**Figure 1:**
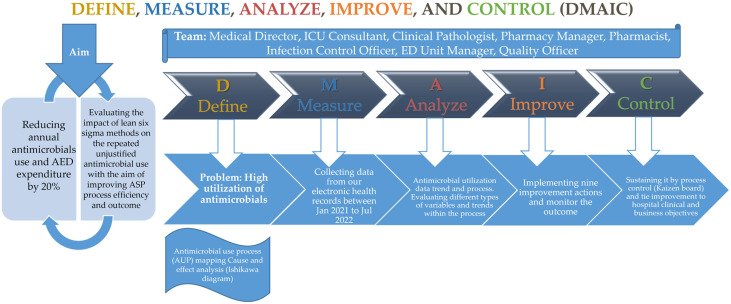
Quality improvement project at MWEL (Mediclinic Welcare Hospital) to improve patient safety through antimicrobial stewardship program (ASP) using the Lean Six Sigma (LSS) methodology via the Define, Measure, Analyze, Improve, and Control (DMAIC) approach. AED: the United Arab Emirates Dirham; ICU: intensive care unit; ED: emergency department.

The LSS initiative was designed and implemented by employing the DMAIC project development approach, as shown in (Figure 1).

**Figure 2:**
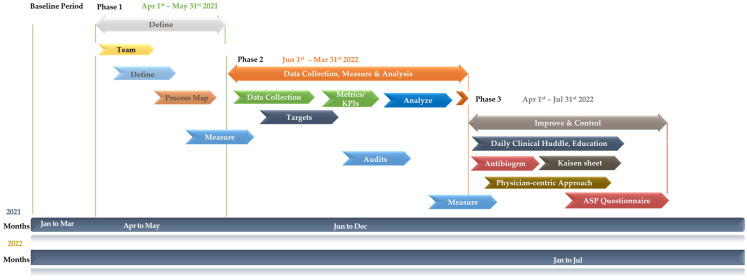
Gantt chart illustrating the timelines and key steps used in the antimicrobial stewardship Pprogram (ASP) improvements at MWEL (Mediclinic Welcare Hospital). KPIs: key performance indicators.

A Gantt chart was used as the study planning tool for visualizing and monitoring the progress of tasks over time [41]. Essential tasks were identified with time frames and relationships between the tasks. As illustrated in (Figure 2), the Gantt chart includes the project start date, description of each project phase, start and end dates for every phase, and project end date. The intervention period was further divided into three phases: the Define phase (1 April 2021–31 May 2021), the Measure/Analyze phase (1 June 2021–31 March 2022), and the Improve/Control phase (1 April 2022–31 July 2022).

### 2.2. Ethics Statement

The study was conducted in accordance with the Declaration of Helsinki. Ethical approval was obtained from the MCME Research and Ethics Committee (REC) (No. MCME.CR.277.MWEL.2022), issued on 5 October 2022. In addition, the study was approved by the Dubai Scientific and Research Ethics Committee (DSREC) at Dubai Health Authority (DHA) (No. DSREC-02/2023_5), issued on 21 February 2023.

### 2.3. Descriptive Analysis of the Nine ASP Interventions

Due to the inherent complexity and multifaceted nature of these ASP interventions, rendering their direct quantification inadequate for capturing their full impact, a qualitative description was used for the assessment of the nine distinct ASP interventions in this study as follows:

1. Leadership support: descriptive assessment of the involvement of leadership personnel to advocate prudent antimicrobial use practices.
2. Guidelines for antimicrobial use: descriptive assessment of the guidelines established to outline proper and prudent antimicrobial drug usage.
3. Formulary restriction with prior authorization: assessment of the imposed restrictions on the availability of specific antimicrobials through formulary control, requiring prior authorization for prescription.
4. Utilization audits: descriptive assessment of antimicrobial prescribing patterns and practices.
5. Utilization of local antibiogram and adherence to culture and susceptibility results: descriptive assessment of local antibiograms and adherence to culture and susceptibility data to guide antimicrobial selection.
6. Conversion from IV to PO antimicrobials: assessment following the patients transitioning from IV to PO antimicrobial administration when clinically appropriate.
7. Assessment of antimicrobial use (selection, dosage, route, and duration): assessed through evaluation of antimicrobial therapy, considering appropriateness in terms of drug selection, dosage, route of administration, and duration of treatment.
8. Feedback/education: assessment of the constructive feedback and educational resources provided for healthcare practitioners to enhance their understanding of optimal antimicrobial prescribing practices.
9. Proper cleaning and hand hygiene: assessment of effective cleaning practices and proper hand hygiene adherence.

### 2.4. Assessment of the CTQs

The six CTQs included: (1) total expenditure on antimicrobials; (2) antimicrobial consumption per unit; (3) DDD per 100 bed-days; (4) surgical prophylaxis appropriateness; (5) average length of stay; and (6) frequency of clinical pharmacist ASP intervention.

The four surgical prophylaxis appropriateness criteria were as follows: (1) Criterion 1: preoperative antibiotic prophylaxis—right drug (as per antibiotic prophylaxis guidelines); (2) Criterion 2: preoperative antibiotic prophylaxis—right time (0–60 min before incision); (3) Criterion 3: preoperative antibiotic prophylaxis—right dose, as per prophylaxis guidelines; (4) Criterion 4: antibiotic stopped within 24 h after surgeries. 

### 2.5. Data Analysis 

Data analyses were conducted using IBM SPSS Statistics for Windows, Version 29.0. (Armonk, NY, USA: IBM Corp). The two-tailed Mann–Whitney *U* test (M-W) and the Kruskal–Wallis (K-W) test were used to assess the associations between scale and categorical variables.

The presence of a linear trend in the association of the study variables was assessed using the linear-by-linear test for association (LBL). The cutoff for statistical significance was set at *p* = 0.050

## 3. Results

### 3.1. Description of the Study Phases

The study comprised a total of 19 consecutive months starting on 1 January 2021 and ending on 31 July 2022. The description of the average monthly expenditure in AED and units, as well as the average defined daily dose (DDD) per 100 bed-days and admissions stratified by the study phase is shown in (Table 1).

**Figure 3:**
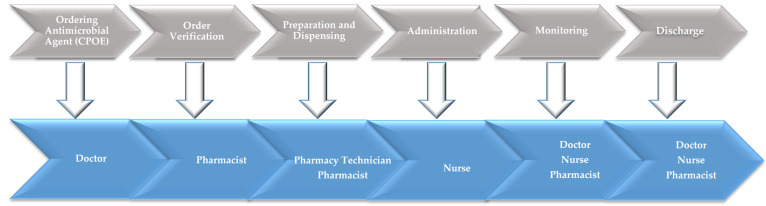
Antimicrobial use process (AUP) mapping and the possible healthcare professionals that are involved at each step.

With the problem statement “excess rates of antimicrobial agents use and cost expenditures” and objectives of the project, the ASP multidisciplinary work group “Medical Director, Pharmacy Manager, Intensivists, Clinical Pathologist, Clinical Pharmacist, Infection Control Officer, Nursing Department Unit Manager, Quality Officer” constructed a process map to define each step in the actual process and the possible healthcare professionals who are involved at each step (Figure 3).

**Figure 4:**
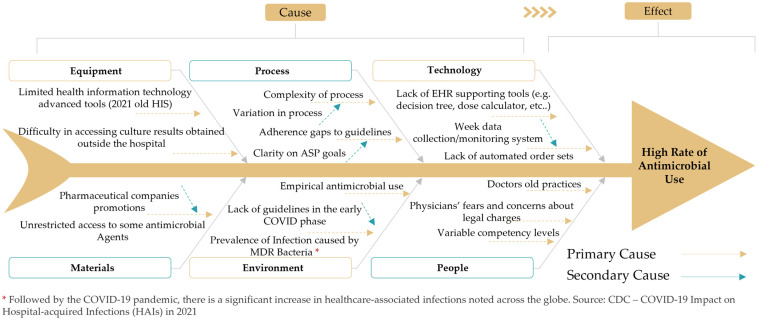
Fishbone (Ishikawa) diagram identifying all possible factors leading to excess rates of parenteral antimicrobial agent use.

Subsequently, the team conducted a cause-and-effect analysis (Ishikawa diagram) to identify all possible factors leading to excess rates of antimicrobial agent use (Figure 4). A targeted approach has been adopted to combat antimicrobial misuse, with interventions precisely aligned to the identified causes in the Ishikawa diagram (Table 2). Critical interventions included leadership support, which targeted the “People” and the “Process” aspects, to enhance adherence to guidelines and to raise the culture of accountability. The formulation of antimicrobial use guidelines addressed the “Materials”, “Process”, and “Technology” aspects to ensure the prudent prescribing of antimicrobials. Formulary restrictions and utilization audits addressed the “Process” factor, promoting compliance with guidelines and effective oversight of the program. Interventions such as utilizing local antibiograms, conversion from IV to PO antimicrobials, and feedback/education addressed various other factors, including ”Equipment”, “Materials”, and “People”. Additionally, measures for proper cleaning and hand hygiene were directed to the environmental aspects, in order to mitigate infection spread.

This systematic approach, which aligned each intervention with the specific causes as identified in the Ishikawa diagram, demonstrated a targeted strategy to reduce antimicrobial use at MWEL.

### 3.2. Descriptive Assessment of ASP Interventions over the Intervention Phases 

A full description of the steps taken to improve the antimicrobial stewardship program divided into every step of the nine interventions is shown in (Table 3).

Revitalizing the ASP decreased hospital-wide parenteral antimicrobial expenditures in AED (Emirati Dirham) between January and July of 2021 and 2022 by 81.7%, while preserving the same clinical outcomes (Table 1). The expenditure in AED for each parenteral antimicrobial agent is shown in (Table S1).

Additionally, ASP interventions decreased hospital-wide parenteral antimicrobial usage (consumption) by 54.2%, while preserving the same clinical outcomes. The average percentage change in the defined daily dose across the seven months of January to July 2021 vs. 2022 was approximately 12.5%, indicating a general reduction in medication consumption in 2022.

Moreover, ASP interventions reduced both high-risk agent AED costs (injectable fluoroquinolones (ciprofloxacin, levofloxacin, and moxifloxacin) and 3rd generation cephalosporins (ceftriaxone, cefotaxime, and ceftazidime)). The data in (Table S2) highlights the reductions in antimicrobial spending for fluoroquinolones and 3rd generation cephalosporins during the study period, indicating the ASP impact on cost management. Fluoroquinolone costs dropped by 53.2%, from AED 149,912 to AED 70,137, while 3rd generation cephalosporin expenses reduced by 51.2%, from AED 322,289 to AED 157,318 (Table S2). 

From September 2021, the list of restricted antimicrobial agents was reviewed and updated to include the following: carbapenems (meropenem and ertapenem), combinations of penicillin with beta-lactamase inhibitors (piperacillin–tazobactam), combinations of fifth-generation cephalosporin with beta-lactamase inhibitors (ceftolozane–tazobactam), combinations of third-generation cephalosporins with beta-lactamase inhibitors (ceftazidime–avibactam), fourth- and fifth-generation cephalosporins (cefepime and ceftobiprole), glycopeptides (vancomycin and teicoplanin), polymyxins (colistimethate), oxazolidinones (linezolid), glycylcyclines (tigecycline), enchocandin antifungals (caspofungin and anidulafungin), triazole antifungals (fluconazole, voriconazole, and isavuconazole), and antimycotics antifungals (amphotericin B liposomal). The percentage of patients receiving restricted antimicrobial agents per admission decreased over the study period from 5.25% to 2.22%, remaining below the 10% target throughout September 2021 to July 2022 (Table S3).

### 3.3. Critical-to-Quality Improvement following Interventions 

Stratified per study phase, the total expenditure on antimicrobials significantly dropped from a monthly mean of AED 661,680 in the baseline phase to a mean of AED 139,752 in the ASP intervention phase (*p* = 0.008, M-W). Considering the baseline phase and stratification for each of the three phases of the DMIAC method, the expenditure dropped from a monthly mean of AED 417,320 in the Define phase to a mean of AED 105,240 in the Measure/Analyze phase, and finally a monthly mean of AED 87,249 in the Improve/Control ASP intervention phase (*p* = 0.013, K-W).

For the antimicrobial consumption per unit, a significant drop was observed from a monthly mean of 5597 units in the baseline phase to a monthly mean of 2701 units in the ASP intervention phase (*p* = 0.008, M-W). Additionally, the consumption per unit decreased significantly over the intervention phases with a mean of 4298 units in the Define phase to a mean of 2624 in the Measure/Analyze phase, and finally a monthly mean of 2093 in the Improve/Control phase (*p* = 0.015, K-W).

The DDD per 100 bed-days dropped from 2.42 in the baseline phase to 1.77 in the intervention; however, this drop lacked statistical significance (*p* = 0.064, M-W). The same pattern was observed over the four phases, with a mean of 2.16 in the Define phase, to 1.70 in the Measure/Analyze phase and 1.76 in the Improve/Control phase (*p* = 0.102, K-W).

For the surgical prophylaxis appropriateness criteria, the compliance increased from 68% in January 2022 to 88% in July 2022 (*p* = 0.077, LBL, Table S4).

The average length of stay was reduced significantly from 4.18 days in July 2021 to 1.40 days in July 2022 (*p* = 0.004, LBL). The clinical pharmacist ASP intervention frequency (de-escalation, dose optimization, and discontinuation) also showed a steady increase, from 19 interventions in April 2022 to a total of 45 interventions in July 2022 (*p* = 0.087, LBL, Table S5).

## 4. Discussion

The current study results demonstrate the utility of the LSS methodology in addressing the crucial challenge of AMR within the framework of ASPs. While several investigations have showed the effectiveness of applying the LSS methodology in healthcare, particularly with a concentrated emphasis on ASPs, our results within the UAE context mark a novel contribution [21,22,23,42]. In this study, we addressed the primary causes of excessive parenteral antimicrobial use, including pharmaceutical promotions, unrestricted access to antimicrobials, empirical and outdated prescribing practices, and the prevalence of MDR bacteria through a comprehensive, evidence-based ASP. The interventions were based on securing leadership commitment, allocating essential resources, establishing robust communication channels, forming an interdisciplinary ASP team, continuously updating evidence-based guidelines, and introducing a user-friendly electronic platform for guideline dissemination. These strategic actions collectively aimed to mitigate the root causes of excessive antimicrobial use and promoted evidence-based prescribing practices.

As indicated by the study results, the areas of improvement included the marked reduction in expenditure on the antimicrobial agents and reduction in the consumption of antimicrobials. Specifically, the analysis revealed significant reductions in expenditures, with an 81.7% decrease from January 2021 to July 2022, highlighting the cost-containment impact of LSS. Examining the DDDs revealed a 12.5% average reduction in utilization during 2022, indicating interventions optimizing antimicrobial usage.

Previous studies showed the impact of LSS in reducing costs in healthcare practice as follows [43,44]: An early study at the University Medical Center Groningen, Netherlands, showed a reduction in the cost of surgical implants following the implementation of the LSS approach [45]. Another study that assessed the impact of LSS on the length of stay among patients on prolonged mechanical ventilation at an academic medical center in New Jersey, the US, highlighted the significant cost reductions [46].

Regarding the utility of LSS in ASP intervention involving the proper use of antimicrobials, the current study focused on high-risk agents, namely injectable fluoroquinolones and 3rd generation cephalosporins. The results demonstrated the reduced average expenditure costs and utilization due to ASP interventions.

Utilizing advanced metrics, the study assessed restricted antimicrobial utilization in hospital wards to evaluate the ASP’s impact on reducing usage and costs. Our analysis revealed fluctuations in the percentage of patients receiving at least one restricted antimicrobial agent per admission, ranging between 2% and 5.3% across different intervention months. Despite the initial target being set at 5%, it is noteworthy that the observed percentages consistently remained well below the established 10% threshold. This observation suggests the proper adherence to the protocols outlined by the ASP, thereby achieving successful management of the utilization of restricted antimicrobial agents. In a related context, a study from Mount Sinai Medical Center, New York, the US, showed the value of the LSS methodology in reducing excess dosing in patients with renal disease [47].

In assessing the utilization rate of restricted antimicrobial agents relative to the overall utilization of inpatient medications, we identified a variable range from 1.2% to 8.1%, with the target rate set at 10%. This analysis emphasized the program’s effectiveness in controlling the utilization of restricted antimicrobial agents, although certain months did exhibit utilization rates exceeding the predetermined target. 

In the pursuit of continuous improvement using the LSS methodology, it is important to recognize that achieving desired outcomes is just one aspect of the equation. Ensuring sustainability of the improvements necessitates a proactive approach involving ongoing education and monitoring [48,49,50]. In this study, we emphasize the significance of continuous learning with a key intervention measure involving the regular updating of the guidelines based on the latest clinical evidence and emerging AMR patterns. Ongoing education empowers healthcare professionals with knowledge and skills for effective guideline implementation, data-driven decision making, and adaptation to changing circumstances [51]. Furthermore, consistent monitoring and KPI measurement are integral to the LSS methodology [21]. Thus, the commitment to ongoing education and vigilant monitoring should be highlighted as an essential measure for the enduring success and sustainability of improvements in ASP [52].

We further explored utilization rates across distinct MWEL units, including the ICU, medical and surgical wards, pediatric ward, neonatal ICU, and maternity ward. The results demonstrated that the ICU was consistently showing the highest utilization of restricted antimicrobial agents. This observation underscores the critical importance of tailored stewardship strategies, especially within the context of ICUs, particularly during times of pandemics or heightened AMR concerns. In the context of the COVID-19 pandemic, there have been notable global concerns regarding the increased consumption of antimicrobials and the potential emergence of AMR [53,54,55]. Several recent studies reported an increase in antimicrobial usage during the COVID-19 pandemic due to various factors, including increased hospitalizations and heightened concerns about secondary infections [55,56,57,58]. Nevertheless, a recent study conducted in the UAE shed light on the adaptability of ASP teams and services in the country during the pandemic, with findings indicating the ability of ASP team members in the UAE to repurpose their roles to respond effectively to the unique COVID-19 challenges [59]. This adaptability was demonstrated through the involvement in developing national guidelines for the treatment of COVID-19 patients and the active contribution to guideline management and monitoring [59]. Despite the initial disruptions caused by the pandemic, a gradual restoration of ASP activities was reported by Hashad et al. in the UAE, highlighting the resilience and commitment of healthcare professionals in maintaining ASP activities [59,60].

In this study, compliance with best-practice surgical prophylaxis bundles was scrutinized, identifying notable noncompliance in emergency lower segment caesarean section (LSCS) procedures, specifically regarding prophylaxis timing. The ASP interventions effectuated enhancements in compliance, and the assessment encompassed appropriateness and discontinuation of antimicrobial surgical prophylaxis within 24 h post-surgery.

Evaluating antimicrobial prophylaxis appropriateness and timely cessation within 24 h post-surgery was pivotal. The criteria included the drug, timing, dosage per guidelines, and cessation timing. Varied compliance and appropriateness emerged, accentuating dynamic guideline adherence. Continued vigilance is pivotal for sustained compliance and patient safety. Additionally, transitioning from an IV to PO administration, pivotal for antimicrobial therapy optimization, was implemented. Monitoring the IV-to-PO switch compliance revealed a commendable 96% rate increase by July 2022, enhancing patient-centered care and cost efficiency.

Compliance to hand hygiene, which is considered of paramount importance for reducing healthcare-associated infections, improved to 91% by July 2022. Effective cleaning and disinfection, crucial for pathogen control, align with persistent environmental threats. Administering 4% chlorhexidine gluconate (CHG) baths exhibited success in mitigating surgical site infections. Pathogen persistence underscores the stringent cleaning validation protocols’ significance. The positive impact of LSS in adherence to hand hygiene, with a subsequent reduction in hospital-acquired MRSA and reduction in costs, was highlighted in an early study at the Presbyterian Healthcare Services in Albuquerque, New Mexico [61].

### 4.1. Study Strengths and Recommendations Based on the Study Findings

The current study findings could pave the way for further research to highlight the role of LSS as an important methodology for quality improvement in healthcare with subsequent positive outcomes. Future studies at MWEL are recommended to focus on the long-term sustainability of the improvements achieved through the LSS-based ASP interventions. Additionally, comparative studies involving multiple healthcare facilities could offer insights into the generalizability and scalability of LSS in the context of ASPs. Further research and ongoing commitment to evidence-based practices are essential to building upon the successes observed in this study and addressing the global challenge of AMR.

The findings of this study could be beneficial to show the positive impact of quality improvement approaches in clinical practice. The study results highlighted that a well-executed ASP utilizing the LSS methodology could substantially reduce antimicrobial expenditure, improve compliance with best practice guidelines, shorten hospital stays, and improve the patient-centered care. Healthcare facilities could use our findings as a model for optimizing antimicrobial use; hence, our study results could contribute to the global effort aiming to combat AMR.

### 4.2. Study Limitations

Finally, despite the value of the findings of the current study, several limitations should be explicitly mentioned. These limitations, which must be carefully considered when interpreting the results of our study, included: (1) The retrospective design implemented in this study has inherent limitations, including the reduced ability to capture the dynamic nature of AMR and the patterns of antimicrobial use. Additionally, the retrospective data can be affected by selection bias and recall bias, which may limit the ability to establish cause-and-effect relationships. (2) The incomplete data could have resulted in biased analysis, limiting the generalizability of the study results. (3) The effectiveness of the LSS interventions in ASPs requires a long-term evaluation to meticulously delineate the significant changes in patterns and trends. Thus, the limited timeframe employed in this study, albeit helpful over the short term, may not provide a comprehensive view of the impact of LSS interventions. Therefore, future studies are recommended to assess the long-term benefits of implementing LSS in ASPs at MWEL among other healthcare centers. (4) The current study was conducted at a single center, which may limit the generalizability of the findings. This limitation is related to the different and unique patient populations, antimicrobial prescribing practices, and AMR patterns at various healthcare centers. Therefore, the results of an LSS-based intervention at a single healthcare center might not be generalizable to other settings, with subsequent need to conduct further multi-center studies. (5) The awareness of health professionals at MWEL regarding the implementation of the LSS interventions could have led to a phenomenon known as the Hawthorne effect, where individuals modify their behavior in response to their awareness of being observed [62]. In the context of ASPs, this could mean that health professionals altered their prescribing habits due to the prior knowledge that their actions are being monitored and studied. This can introduce a significant bias in the study, as the observed changes may not be reflective of genuine improvements in the prescribing practices.

## 5. Conclusions

This study highlighted the importance of implementing LSS methodology as a valuable approach in healthcare practices, enhancing the outcomes of ASPs and reducing the overall antimicrobial usage and its associated costs. The findings could have significant implications for healthcare policymakers seeking to enhance future ASP outcomes through evidence-based strategies.

## Figures and Tables

**Table 1:** Description of key study parameters stratified per study phase.

Study Phase	Duration	Mean Expenditure in AED ^1^	Mean Expenditure in Units	Mean DDD ^2^ per 100 Bed-Days	Mean Admissions
Baseline	1 January 2021–31 March 2021	661,680	5597	2.417	616
Define	1 April 2021–31 May 2021	417,320.5	4298	2.155	723.5
Measure/Analyze	1 June 2021–31 March 2022	105,240.5	2624.2	1.704	709.9
Improve/Control	1 April 2022–31 July 2022	87,249	2092.75	1.758	791.75

^1^ AED: the United Arab Emirates Dirham; ^2^ DDD: defined daily dose per 100 bed-days.

**Table 2:** Interventions implemented to address the possible factors leading to excess rates of parenteral antimicrobial agent use identified through the Ishikawa diagram.

Cause Identified from Ishikawa Diagram	Corresponding Intervention
Limited health information technology tools	Utilization of local antibiogram and adherence to culture and susceptibility results; guidelines for antimicrobial use
Difficulty in accessing laboratory microbiologic culture results	Utilization of local antibiogram and adherence to culture and susceptibility results
Unrestricted access to some antimicrobial agents	Guidelines for antimicrobial use; formulary restriction with prior authorization
Complexity in process	Formulary restriction with prior authorization; conversion from IV to PO antimicrobials
Variation in process	Utilization audits; assessment of antimicrobial use
Adherence gaps in guidelines	Leadership support; feedback/education
Clarity on ASP goals	Leadership support; utilization audits
Empirical antimicrobial use	Guidelines for antimicrobial use; feedback/education
Lack of guidelines in the early COVID-19 phase	Feedback/education
Lack of EHR supporting tools	Guidelines for antimicrobial use
Weak data collection/monitoring system	Utilization audits
Lack of automated order sets	Guidelines for antimicrobial use
Doctors’ old practices	Feedback/education
Variable competency levels	Utilization audits; feedback/education
Physicians’ fears and concerns about legal charges	Leadership support

ASP: antimicrobial stewardship; COVID-19: coronavirus disease 2019; EHR: electronic health record; IV: intravenous; PO: oral.

**Table 3:** Description of the interventions used to improve antimicrobial stewardship program (ASP).

Intervention	Measures Taken
Leadership support	The multidisciplinary team for the ASP at MWEL includes senior leaders from the hospital, such as the medical director, pharmacy manager, quality officer, and other key physicians and unit managers. These individuals possess the necessary expertise and skills to address significant shortcomings and effectively train their peers on ASP guidelines, promoting adherence to best practices. Also, MWEL leadership demonstrated a commitment to the requisite human, financial, and information technology resources essential for the success of our ASP initiative.
Guidelines for antimicrobial use	Allocated necessary resources, personnel, and budget to support the implementation of ASP strategies. Data comparisons of antimicrobial use before and after the introduction of the guidelines to measure their effectiveness.
Formulary restriction with prior authorization	Established clear communication channels for disseminating ASP goals, progress, and achievements
Utilization audits	Established a dedicated ASP team comprising interdisciplinary members to oversee program implementation
Utilization of local antibiogram and adherence to culture and susceptibility results	Conducted regular meetings to update hospital leadership on ASP progress, challenges, and achievements
Conversion from IV to PO antimicrobials	Encouraged hospital executives to actively participate in ASP-related activities and initiatives
Assessment of antimicrobial use (selection, dosage, route, and duration)	Developed evidence-based guidelines for appropriate antimicrobial prescribing and usage
Feedback/education	Ensured guidelines were regularly updated based on the latest clinical evidence and emerging resistance patterns
Proper cleaning and hand hygiene	Created a user-friendly electronic platform (Intranet) for easy access to and dissemination of the antimicrobial guidelines to all clinical staff

## Data Availability

The data that support the findings of this study are available from the corresponding author (M.S.) upon rational request.

## Supplementary Materials

The following supporting information can be downloaded at: https://www.mdpi.com/article/10.3390/healthcare11233048/s1, Table S1: Data for AED expenditures per each parenteral antimicrobial agent collected between Jan 2021 to July 2022; Table S2: Monthly DDD/100 Bed days for each Injectable antimicrobial agent during the study period; Table S3: Number of patients received at least one restricted antimicrobial agent per admission vs. total number of IP admitted patients; Table S4: Surgical Prophylaxis Appropriateness; Table S5: Clinical pharmacist ASP intervention frequency (De-escalation, Dose optimization, Discontinuation).

## Abbreviations

AED	The United Arab Emirates Dirham
AMR	Antimicrobial resistance
ASP	Antimicrobial stewardship program
AUP	Antimicrobial use process
CDC	The Centers for Disease Control and Prevention
CHG	Chlorhexidine gluconate
CTQ	Critical-to-quality
DDD	Defined daily dose
DMAIC	Define, Measure, Analyze, Improve, Control
ED	Emergency department
ESBL	Extended spectrum beta-lactamase
ICU	Intensive care unit
ID	Infectious disease
IV	Intravenous
JCI	Joint Commission International
KPI	Key performance indicator
K-W	Kruskal–Wallis
LBL	Linear-by-linear test for association
LSS	Lean Six Sigma
MDR	Multidrug resistant
MRSA	Methicillin-resistant Staphylococcus aureus
M-W	Mann–Whitney U test
MWEL	Mediclinic Welcare Hospital
PO	Oral
UAE	The United Arab Emirates
VRE	Vancomycin-resistant Enterococcus
