# Supplementary material for: Improving Antimicrobial Stewardship Program Using the Lean Six Sigma Methodology: A Descriptive Study from Mediclinic Welcare Hospital in Dubai, the UAE
Source: Healthcare (Basel). 2023 Nov 27;11(23):3048. doi: 10.3390/healthcare11233048 (PMC10706105; doi:10.3390/healthcare11233048)
Supplement: Supplementary file 1 [file healthcare-11-03048-s001.zip › healthcare-2667297-supplementary.pdf]

Table S1. Data for AED expenditures per each parenteral antimicrobial agent collected between Jan 2021 to July 2022.

AMR agent	2021 AED expenditures	AMR agent	January–July 2022 AED expenditures
Aciclovir	26,410	Aciclovir	5,437
Amikacin	5,455	Amikacin	1,240
Amoxicillin/potassium clavulanate	13,528	Amoxicillin/potassium clavulanate	5,142
Amphotericin B liposome	38,089	Amphotericin B liposome	0
Ampicillin	15,369	Ampicillin	5,562
Anidulafungin	8,821	Anidulafungin	0
Azithromycin	19,253	Azithromycin	16,459
Benzathine benzylpenicillin	0	Benzathine benzylpenicillin	59
Caspofungin	1,367,605	Caspofungin	60,370
Cefazolin	22,677	Cefazolin	7,973
Cefepime	12,181	Cefepime	3,499
Cefotaxime	12,296	Cefotaxime	13,001
Ceftazidime	30,941	Ceftazidime	900
Ceftazidime/avibactam	266,634	Ceftazidime/avibactam	29,520
Ceftobiprole medocaril	6,691	Ceftobiprole medocaril	5,282
Ceftriaxone	446,635	Ceftriaxone	5,358
Cefuroxime	47,279	Cefuroxime	144,318
Ciprofloxacin	104,570	Ciprofloxacin	23,726
Colistimethate	2,032	Colistimethate	48,306
Doxycycline	419	Doxycycline	1,355
Ertapenem	46,587	Ertapenem	35,989
Fluconazole	62,320	Fluconazole	7,133
Gentamicin	7,565	Gentamicin	1,143
Isavuconazole	215,573	Isavuconazole	0
Levofloxacin	90,407	Levofloxacin	16,907
Linezolid	121,881	Linezolid	4,163
Meropenem	293,478	Meropenem	35,034
Metronidazole	37,494	Metronidazole	17,157
Moxifloxacin	8,059	Moxifloxacin	4,925
Piperacillin/tazobactam	120,558	Piperacillin/tazobactam	26,220
Sulfamethoxazole/trimethoprim	2,610	Sulfamethoxazole/trimethoprim	0
Teicoplanin	2,517	Teicoplanin	719
Tigecycline	72,108	Tigecycline	20,664
Vancomycin	74,928	Vancomycin	8,262
Voriconazole	53,320	Voriconazole	8,967

Table S2. Monthly DDD/100 Bed days for each Injectable antimicrobial agent during the study period.

Injectable Antimicrobial	21-Jan	21-Feb	21-Mar	21-Apr	21-May	21-Jun	21-Jul	21-Aug	21-Sep	21-Oct	21-Nov	21-Dec	22-Jan	22-Feb	22-Mar	22-Apr	22-May	22-Jun	22-Jul
Aciclovir (Acyclovir)	0.16	0	0	0.02	0.07	0.22	0.25	0.44	0.03	0.13	0	0.68	0.04	0.01	0.18	0.04	0	0.25	0.06
Amikacin Sulfate	0.1	0.26	1.24	0.37	0	0.6	0.6	0.4	0.26	0	0.22	0.07	0.04	0.57	0.42	0	0.15	0.57	0
Amoxicillin, Potassium Clavulanate	2.22	1	2.21	2	1.99	1.81	1.61	1.84	1.57	1.25	1.74	2.5	1.9	2.3	1.38	2.55	1.46	1.73	2.68
Amphotericin B (Liposomal)	0	0	0	3.49	1.43	0	0	0	0	0	0	0	0	0	0	0	0	0	0
Ampicillin	0.87	1.14	1.18	2.3	1.81	0.4	1.38	2.54	0.96	0.94	0.63	1.06	1.66	1.56	0.94	0.9	2.1	0.72	1.18
Anidulafungin	0	0	0	0.29	0.4	0	0	0	0	0	0	0	0	0	0	0	0	0	0
Avibactam, Ceftazidime	0	0.78	2.63	1.94	0.8	0.24	1.14	0.07	0.73	0	0.13	0	0	0	0	0.72	0.1	0.27	0.8
Azithromycin (Dihydrate)	1.38	1.07	1.35	0.93	2.01	1.55	2.51	2.25	2.28	2.75	4.65	2.81	2.5	2.7	4.05	7.37	3	4.1	4.72
Benzathine Penicillin	0	0	0	0	0	0	0	0	0	0	0	0	0	0.09	0	0	0	0	0
Caspofungin	0.35	9.81	7.15	6.26	2.16	0.68	0.96	0.27	3.09	0.67	0	0	0.55	1.74	0	0.38	0.85	2.22	0
Cefazolin Sodium	0	0	0.01	5.51	7.9	6.32	6.25	6.66	6.59	5.66	5.36	5.83	5.71	6.54	6.48	8.32	6.54	5.2	8.07
Cefepime Hydrochloride	0	1.23	0.77	0.4	0.04	0	0.94	0.19	0	0	0.18	0.56	0.29	0.39	0.57	0.47	0	0.29	0.23
Cefotaxime Sodium	0.52	0.5	0.09	0.23	0.05	0.17	0.64	0.12	0.86	0.73	1.04	0.41	1.37	0.78	0.7	0.4	1.48	2.05	0.23
Ceftazidime Pentahydrate	0.41	0.58	3.91	3.55	0.16	0.1	0.27	0.11	0.29	0.46	0.78	0	0	0.09	0.04	0	0.5	0	0
Ceftobiprole	0	0.14	0.3	0	0	0	0	0	0	0	0	0	0	0	0	0	0	0.61	0
Ceftolozane Sulfate, Tazobactam Sodium	0	0	0	0	0	0	0	0	0	0	0	0	0	0	0	0	0	0	0.44
Ceftriaxone Sodium	32.3	24.77	19.74	15.55	16.06	17.59	16.34	14.52	22.56	14.43	14.71	26.75	17.08	18.53	18.33	18.34	18.17	19.72	18.93
Cefuroxime Sodium	1.22	0.61	1.28	2.13	2.9	3.53	3.65	3.53	3.25	6.27	4.4	4	3.56	2.55	1.94	6.47	4.52	6.48	2.31
Ciprofloxacin	0	0	0	2.2	3.84	0.58	1.86	1.77	0.7	1.22	1.6	1.8	2.33	1.13	3.76	2.88	2.12	1.68	1.98
Colistimethate Sodium	0	0	0	0	0	0	0	0	0	0	0	0	0	0	0	0	0	0	0
Cotrimoxazole	0.08	0.43	0.24	0.37	0	0	0	0.43	0	0	0	0	0	0	0	0	0	0	0
Doxycycline	0	0	0.75	0	0	0	0	0	0	0	0	0	0	0	0	0	0	0	0
Ertapenem Sodium	0.42	0.51	1.62	0.44	0.65	0.34	0.29	0.81	0.81	1.06	1.01	2.66	3.13	2.7	1.01	4.54	1	0.74	0.8
Fluconazole	0.23	4.56	5.01	3.57	3.11	1.31	1.73	0.27	1.76	0.34	1.06	0	0.78	0.44	0.17	2.08	0.46	0.81	0
Gentamicin Sulfate	1.55	4.91	7.93	6.34	6.68	3.92	4.1	3.45	1.52	2.11	3.06	2.18	2.14	2.96	2.84	2.68	3.98	2.53	4.92
Isavuconazole	0.69	0.85	3.27	0	0	0	0	0	1.4	0.06	0	0	0	0	0	0	0	0	0

Levofloxacin Hemihydrate	8.21	11.43	9.26	4.45	2.81	4.79	5.06	9.61	1.1	1.85	2.91	2.95	3.21	2.44	4.22	2.36	2.77	2.76	4.12
Linezolid	0	3.2	7.3	3.13	0.05	0	1.16	4.4	2.65	0	0.11	0.14	0.47	0	0	0.76	0.15	1.28	0
Meropenem	4.36	14.73	11.15	8.67	2.89	4.16	7.53	17	9.99	5.96	1.27	1.54	3.62	5.96	2.31	1.67	3.44	1.23	1.17
Metronidazole	3.13	1.28	2.66	3.34	3.51	2.4	3.61	4.81	3.38	3.88	3.81	2.38	3.47	5.11	2.62	5.32	2.87	2.04	3.55
Moxifloxacin Hydrochloride	0	0	0.45	0.59	0	0	0.63	0	0	0.06	0.56	0.43	0	0.87	0.17	0.19	1.08	0.27	0.3
Piperacillin Sodium, Tazobactam Sodium	0.89	5.74	7.55	4.23	2.67	0.69	2.52	2.12	4.64	1.47	0.74	1.11	4.67	0.27	1.95	2.19	0.9	3.52	1.89
Teicoplanin	0	0	0	0	0.15	0	0.05	0.43	0	0	0.11	0	0	0	0.25	0	0	0	0.1
Tigecycline	0	0	1.11	4.01	0.98	0.48	0	0.43	1.36	0	0	0	1.06	0.04	0.25	0.28	0.81	0.27	0.35
Vancomycin Hydrochloride	2.67	4.06	4.29	1.21	0.72	0.52	0.95	1.06	2.35	1.15	0.71	0.31	0.76	0.63	0.57	0.54	0.79	0.61	0.65
Voriconazole	0	0.62	0.26	1.47	0.28	0	0.14	0.38	0.66	0	0	0	0.35	0	0	0.71	0.15	0.2	0
**Average Monthly DDD**	**1.72**	**2.62**	**2.91**	**2.47**	**1.84**	**1.46**	**1.84**	**2.22**	**2.08**	**1.46**	**1.41**	**1.67**	**1.69**	**1.68**	**1.53**	**2**	**1.65**	**1.73**	**1.65**

Table S3. Number of patients received at least one restricted antimicrobial agent per admission vs total number of IP admitted patients.

Measure	Sep-21	Oct-21	Nov-21	Dec-21	Jan-22	Feb-22	Mar-22	Apr-22	May-22	Jun-22	Jul-22
Number of patients who received at least one restricted antimicrobial agent per admission	35	23	17	18	36	24	21	21	18	26	15
Total number of admitted patients	667	741	668	676	639	672	799	714	864	913	676
Actual	5.25%	3.10%	2.54%	2.66%	5.63%	3.57%	2.63%	2.94%	2.08%	2.85%	2.22%

Table S4. Surgical Prophylaxis Appropriateness.

Month	Criteria 1	Criteria 2	Criteria 3	Criteria 4	Total Compliance	No. of Surgeries
Jan-22	73%	73%	73%	53%	68%	17
Feb-22	85%	85%	85%	15%	68%	13
Mar-22	100%	100%	100%	33%	83%	12
Apr-22	78%	89%	89%	33%	72%	18
May-22	100%	100%	100%	54%	89%	15
Jun-22	96%	96%	96%	21%	77%	24
Jul-22	100%	100%	100%	53%	88%	18

Table S5. Clinical pharmacist ASP intervention frequency (De-escalation, Dose optimization, Discontinuation).

Intervention Type	Apr-22	May-22	Jun-22	Jul-22
De-escalation	3	6	7	10
Dose Optimization	8	12	18	22
Discontinuation	8	6	10	13
Total Interventions	19	24	35	45
